# Incidence and Risk Factors of Neural Tube Defects in Kashan, Central Iran

**Published:** 2015

**Authors:** Ahmad TALEBIAN, Babak SOLTANI, Mojtaba SEHAT, Abolfazl ZAHEDI, Akram NOORIAN, Motahhareh TALEBIAN

**Affiliations:** 1 Department of Pediatrics, Kashan University of Medical Sciences, Kashan, Iran; 2 Department of Community Medicine, Kashan University of Medical Sciences, Kashan, Iran; 3 Student Research Committee, Kashan University of Medical Sciences, Kashan, Iran; 4 Non-Communicable Pediatric Diseases Research Center, Babol University of Medical Sciences, Babol, Iran

**Keywords:** Body mass index, Ethnicity, Neural tube defects, Abortion history, Incidence

## Abstract

**Objective:**

Neural tube defects (NTDs) are the most common congenital defects of central nervous system due to neural tube closure deficit during the third and fourth weeks of gestational age. Our study was performed to detect the incidence and risk factors of NTDs in Kashan, center of Iran.

**Material & Methods:**

This case-control study was done on all pregnancies with NTD affected neonates (n=91) and 209 pregnancies with normal neonates from February 2007 to December 2012 in three hospitals in Kashan, center of Iran. Annual and the mean incidence of NTDs were calculated. Risk factors including neonatal gender, maternal age, gravidity, maternal abortion history, maternal gestational diabetes (GDM), folic acid use, familial marriage, maternal body mass index (BMI), birth season and family history of NTDs were evaluated by interview with mothers. Univariate and multivariate logistic regression were used to analyze the risk factors.

**Results:**

The mean incidence of NTDs was 2.33 per 1000 births. The multivariate analysis indicated that maternal history of abortion (OR: 4.9, CI: 1.9-12.8), and maternal obesity (OR: 5.4, CI: 1.3-21.8) were significantly associated with NTDs.

**Conclusion:**

Maternal history of abortion and BMI were the major risk factors of NTDs.

## Introduction

Neural tube defects (NTDs) may culminate in stillbirth, spontaneous abortion, early infancy fatality or lifelong disability (1). They are a group of congenital defects that encompass spina bifida, anencephaly and encephalocele, which take place during the process of neurulation three to four weeks after conception (2). The mechanism of NTDs encompasses complete or partial fusion defects of the spine, cranial vault or cerebral hemispheres (3). NTDs are the leading cause of central nervous system congenital anomalies and the second most common cause of birth defects following congenital heart disease (3). The incidence rate of NTDs was reported 0.97 per 1000 births in some European countries (4) and 2.88 per 1000 births in north of Iran (5). Multi-factorial etiologies have been shown for NTDs (3). Incidence rate of NTDs differs by genetic (6) and environmental components (7), prior spontaneous abortion (8), short inter-pregnancy interval (9), folate deficiency during pregnancy (10), poor maternal nutrition, maternal ethnicity and BMI (11). Due to substantial different reports about the incidence and risk factors of NTDs in many parts of the world and lack of previous investigation in our district, this study was performed to detect the incidence and associated risk factors of NTDs in Kashan, center of Iran, which may be beneficial for prevention of NTDs and burden of expenses in the future.

## Materials & Methods

This case-control, hospital-based investigation was done on all pregnancies with NTD affected newborns (n=91) and 209 pregnancies with normal neonates during February 2007 to December 2012, selected from three hospitals (Shabihkhani, Shahid Beheshti and Milad), in Kashan, center of Iran. They were selected by their pediatricians and were referred to Shahid Beheshti Hospital. The diagnosis of NTD affected neonates were confirmed by a pediatric neurologist as case group. For each case group, 2 to 3 normal newborns were selected via simple random sampling as control group that got the statistical results more valuable. The case and control groups and their mothers were evaluated in this study. NTDs were defined by International Classification of Disease, 10th revision (ICD-10). Shabihkhani is the largest university affiliated specialized obstetrics and gynecology hospital (100 beds) in Kashan with the rate of more than 3500 deliveries annually. Shahid Beheshti is a university affiliated general hospital (450 beds) with annual rate of deliveries about 1800. It is the referral center of neonatal and neonatal intensive care unit (NICU) in Kashan City. Milad (60 beds) is a private general hospital with about 700 deliveries yearly. Kashan City has a population of 500000 (12). The majority of ethnic groups comprise of Fars and a minority includes Afghan population. An interview was conducted with mothers in both groups by a pediatrician and a questionnaire was filled regarding risk factors for NTDs. Inclusion criteria were all NTD affected newborns and their mothers. Exclusion criteria were the un-cooperative mothers. All mothers were allowed to resign from the study at any time. Risk factors were included season of birth, maternal family history of NTDs in the first degree relatives, BMI of mothers, familial (consanguineous) marriage, folic acid use during first trimester of pregnancy, age group of mothers, sex of newborn, gravidity, maternal history of abortion and gestational diabetes mellitus (GDM). Maternal underweight, normal weight, overweight and obesity were considered as BMIs<18.5, 18.5-24.9, 25- 29.9 and ≥30 respectively (13). Furthermore, number of deliveries and NTD prevalence were calculated annually and finally the total deliveries and the mean incidence of NTDs were estimated during the six-yearperiod. Data were analyzed using SPSS version 16 (Chicago, IL, USA) software. The study was approved by Ethics Committee of Kashan University of Medical Sciences. A written consent was obtained from parents. Quantitative variables were defined by means and standard deviations (SD) and Qualitative variables were defined by frequencies and percents. Independent t-test was used to compare maternal ages between case and control groups. Univariate logistic regression model was used for each of risk factors and if the P value of them were less than 0.2, they were entered into multiple logistic regression model to control the confounders and the results were presented as OR with 95% confidence interval (CI) and P values less than 0.05 were considered significant.

## Results

**Fig 1:**
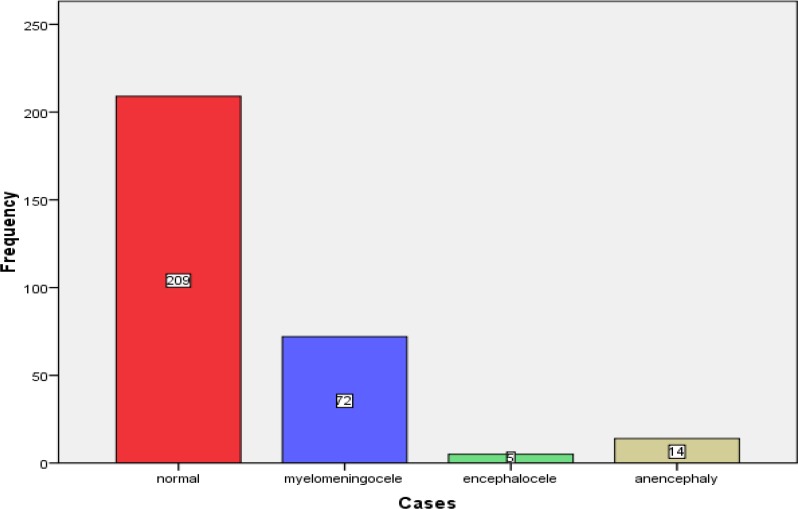
Frequency of cases

During the period of six years, 38936 neonates were born in Kashan City, out of them 91 cases had NTDs with the mean incidence of 2.33 per 1000 births. The most incidence rate was during 2011 (3.62/1000) and the least was during 2012 (1.02/1000). NTDs included spina bifida (79.1%), anencephaly (15.4%) and encephalocele (5.5%). Frequency of cases is indicated in Figure 1. Locations of spina bifida were lumbosacral (62.6%), thoracic (15.4%), lumbar (15.4%) and cervical (6.6%). Totally, the age range of mothers was 14 to 40 yr with the mean age of 27.9±5.8 yr. The mean age of mothers in control group was 28.4±6.1 yr and in case group was 26.7±4.9 yr, respectively (P value=0.012, CI: 0.39-3.03). In case group, 37 (40.7%) affected infants were male and 54 (59.3%) were female. In control group, 101 (48.3%) infants were male and 108 (51.7%) were female (Table 1). Univariate and multivariate analysis of neonatal and maternal characteristics and risk factors for NTDs are depicted in Table 1. Sex of newborns, maternal GDM, folic acid consumption during first trimester of pregnancy, season of birth and maternal family history of NTDs were not significantly associated with NTDs (Table 1). Multivariate analysis indicated that maternal history of abortion (yes/no OR: 4.9, 95% CI: 1.9-12.8) and maternal BMI (obese/underweight OR: 5.4, 95% CI: 1.3-21.8) were significantly associated with NTDs (Table 1).

**Table 1:** Univariate and Multivariate Analysis of Association between Risk Factors and Neural Tube Defects

**Risk factors**	**Case** **n (%)**	**Control** **n (%)**	***P*** ** value**	**Logistic regression**	
				OR (95% CI)	*P* value
Sex					
Male	37 (40.7)	101 (48.3)	-		
Female	54 (59.3)	108 (51.7)	0.22		
Maternal age (yr)					
<20	7 (7.7)	13 (6.2)	-		-
20-35	80 (87.9)	165 (78.9)	0.8		0.94
>35	4 (4.4)	31 (14.8)	0.04		0.059
Gravidity					
1	46 (50.5)	105 (50.2)	-		-
2	22 (24.2)	76 (36.4)	0.17		0.3
3	12 (13.2)	15 (7.2)	0.16		0.2
>3	11 (12.1)	13 (6.2)	0.14		0.96
History of abortion					
No	74 (81.3)	199 (95.2)	-	1	-
Yes	17 (18.7)	10 (4.8)	<0.001	4.9 (1.9-12.8)	0.001
GDM					
No	82 (90.1)	196 (93.8)	-		
Yes	9 (9.9)	13 (6.2)	0.26		
Folic acid use					
No	58 (63.7)	131 (62.7)	-		
Yes	33 (36.3)	78 (37.3)	0.86		
Familial marriage					
No	65 (71.4)	169 (80.9)	-		-
Yes	26 (28.6)	40 (19.1)	0.07		0.4
BMI groups					
Underweight	6 (6.6)	11 (5.3)	-		-
Normal	45 (49.5)	178 (85.2)	0.15		0.2
Overweight	22 (24.2)	13 (6.2)	0.06		0.07
Obese	18 (19.8)	7 (3.3)	0.02	5.4 (1.3-21.8)	0.019
Season of birth					
Spring	28 (30.8)	61 (29.2)	-		
Summer	22 (24.2)	56 (26.8)	0.65		
Autumn	22 (24.2)	41 (19.6)	0.66		
Winter	19 (20.9)	51 (24.4)	0.55		
Family history of NTDs					
No	89 (97.8)	204 (97.6)	-		
Yes	2 (2.2)	5 (2.4)	0.9		

BMI, body mass index; OR, odds ratio; CI, confidence interval; NTDs, neural tube defects; GDM, gestational diabetes mellitus

## Discussion

In our study, the mean incidence of NTDs was 2.33 per 1000 births that was approximately consistent with native Fars ethnicity (2.52 per 1000 births) in northern Iran (5) but lower than north east Iran with 50.1/10000 (14) and 2.97 per 1000 in south east Iran (15). Moreover, our reported incidence of NTDs was higher than the study in England with 17.9/10000 (16) and lower than Turkey with 30.1/10000 (17). These differences may be due to different population studies with their genetic and environmental risk factors. In our research, spina bifida was the most common form of NTDs whereas in the report of Golalipour et al., anencephaly was the most common form (5). We reported no association between neonatal gender and NTDs in contrast to northern Iran with NTDs higher in males (11) and China of NTDs more common in females (18). In another study, spina bifida and anencephaly were more prevalent among females especially greater for anencephaly and the potential explanations included the different survival rate and susceptibility to teratogenic agents between sexes (19). These variations may be due to different genetic, nutrition and other environmental factors between communities. This study presented no association between maternal age group and gravidity with NTDs whereas Bianca et al. showed a U-shaped pattern of association (higher association in lowest and highest age groups and gravidities) (20). As our study, Golalipour et al. indicated no relation between NTDs and gravidity (11). Horn et al. detected that the NTD prevalence was diminished with increase of maternal age (21). During present study, no significant association was detected between seasonality and NTDs whereas Nili et al. reported higher rate of NTDs in births during spring and summer (22). Moreover, summer conceptions were related to higher NTD rate that might be due to more amounts of maternal exposure to solar radiation (23). Afshar et al. reported higher rate of NTDs in pregnancies, which took place in winter (15). Furthermore, they showed that 54% of NTD affected infants had parental consanguinity (15). In Iraq, 63.6% of NTD babies were the product of consanguineous marriage (24). Regarding our results, there was no significant association between NTDs and familial marriage of parents, which was inconsistent with previous studies. The cause of these inconsistencies may be multi factorial. In our study, 36.3% of mothers with NTD infants did not use folic acid during the first trimester of conception. Our findings detected no significant relation between NTDs and consumption of folic acid. Canfield et al. in Texas reported marked reduction of NTD rate by use of folic acid (25), which was incongruous with our study. Similar to our investigation, Golalipour et al. reported no association between folic acid consumption and NTDs (11). An explanation of these different results may be due to various folic acid contents in diets of population in many parts of the world. Based on our results, maternal history of abortion was significantly associated with NTDs (OR=4.9, CI: 1.9- 12.8). According to the research of Golalipour et al., there was no relation between NTDs and history of maternal abortion (11). Our result was discordant to De marco et al. that reported no association between prior spontaneous abortion and NTDs (4). Despite our study, Todoroff et al. presented no association between NTDs and history of previous maternal abortion. It is not known the extent of reported bias or undiagnosed NTDs in prior abortion, which may cause these various associations (26). The majority of newborns with NTDs were born to mothers without history of familial NTDs (27) which was consistent with our study. No association was detected between NTDs and GDM in present study, which was incompatible with Salbaum et al. and the probable cause of it, may be due to high glycemic index as a risk factor of NTDs (28). The present study indicated a significant association between obesity and NTDs that was consistent with Gao et al. which reported obesity as a significant risk factor for NTDs, while underweight and overweight were not recognized as risk factors. The exact mechanism of these different reports is not known but it may be due to variety of diets among pregnant mothers (29). Despite many studies, family history of NTDs and maternal GDM were evaluated in this investigation that was a strong point. Our study has some limitations, we did not evaluate several risk factors such as maternal history of drug use during pregnancy, physical activity/exercise, poor diet (low in vitamins, iron, calcium and high in fat and sugar), dietary folic acid content, B12 use, hot tub using, education of mothers, maternal occupation, socioeconomic status of mothers, interpregnancy interval, smoking and caffeine consumption. Furthermore, we did not check the folic acid serum level during the first trimester of pregnancy, which had more accuracy rather than taking history of folic acid consumption. Furthermore, Afghan population and its control group were insufficient in our study (11 in case group and 8 in control group), so we could not evaluate the ethnicity as a risk factor for NTDs. Finally, further investigations about risk factors for NTDs in aforementioned fields are recommended in the future.

**In conclusion**, maternal history of abortion and obesity were risk factors for NTDs in the present study. Moreover, the incidence of NTDs in our region was substantially higher than some other areas, so further researches with priorities that would increase population-based efforts for determination of NTD risk factors are offered to help the policy makers to make decisions to diminish the NTD prevalence. The data of this research determine the incidence, associated risk factors of neural tube defects, and provide basic information for health care professionals to make policies for implementation of programs to decrease NTD rate and decline the unreasonable costs.
